# Combined expectancies: the role of expectations for the coding of salient bottom-up signals

**DOI:** 10.1007/s00221-019-05710-z

**Published:** 2020-01-13

**Authors:** Michael Wiesing, Gereon R. Fink, Ralph Weidner, Simone Vossel

**Affiliations:** 1 Cognitive Neuroscience, Institute of Neuroscience and Medicine (INM-3), Research Centre Juelich, Wilhelm-Johnen-Strasse, 52428 Juelich, Germany; 2 Department of Neurology, Faculty of Medicine, University Hospital Cologne, University of Cologne, Kerpener Strasse 62, 50937 Cologne, Germany; 3 Department of Psychology, Faculty of Human Sciences, University of Cologne, Gronewaldstrasse 2, 50931 Cologne, Germany

**Keywords:** Probabilistic context, Feature expectancies, Feature binding, Object binding, Prediction error

## Abstract

The visual system forms predictions about upcoming visual features based on previous visual experiences. Such predictions impact on current perception, so that expected stimuli can be detected faster and with higher accuracy. A key question is how these predictions are formed and on which levels of processing they arise. Particularly, predictions could be formed on early levels of processing, where visual features are represented separately, or might require higher levels of processing, with predictions formed based on full object representations that involve combinations of visual features. In four experiments, the present study investigated whether the visual system forms joint prediction errors or whether expectations about different visual features such as color and orientation are formed independently. The first experiment revealed that task-irrelevant and implicitly learned expectations were formed independently when the features were separately bound to different objects. In a second experiment, no evidence for a mutual influence of both types of task-irrelevant and implicitly formed feature expectations was observed, although both visual features were assigned to the same objects. A third experiment confirmed the findings of the previous experiments for explicitly rather than implicitly formed expectations. Finally, no evidence for a mutual influence of different feature expectations was observed when features were assigned to a single centrally presented object. Overall, the present results do not support the view that object feature binding generates joint feature-based expectancies of different object features. Rather, the results suggest that expectations for color and orientation are processed and resolved independently at the feature level.

## Introduction

Perception is not passive and not exclusively determined by the physical properties of sensory stimuli. Rather, it is affected by internal settings such as prior beliefs and probabilistic expectations about upcoming sensory events (Von Helmholtz 1867; Gregory 1997). Prior knowledge in form of experience-based expectancies about behaviorally relevant stimulus features alters how fast and accurately visual stimuli can be detected and perceived. Stimulus features or object properties that are consistent with prior expectations lead to a faster and more accurate stimulus detection, whereas performance is impaired when these features or properties are inconsistent and hence violate current expectations (Dombert et al. 2016b; Kuhns et al. 2017). Expectations not only facilitate stimulus detection (Stojanoski and Niemeier 2015), but also affect object recognition and enhance perceptual sensitivity (Wyart et al. 2012; Stein and Peelen 2015). Such expectations can be induced by either varying the frequency of occurrence of different features in an experiment or by cues that indicate certain features with a specific probability. Rarely occurring or invalidly cued features are assumed to elicit prediction error signals that slow down response times. Compelling evidence for the influence of expectations and prediction error signaling also comes from neuroimaging and electrophysiological studies, in which the perception of unexpected stimuli produces larger neuronal responses than expected ones (Mars et al. 2008; Summerfield and Egner 2009; Kok et al. 2012; Richter et al. 2017; Stefanics et al. 2018).

Behavioral studies suggest that expectations can be formed about single visual features such as color or orientation (Cheadle et al. 2015; Dombert et al. 2016a; Jabar et al. 2017). However, expectations may not only concern single features but also refer to fully integrated object representations. For instance, contextual probabilities can affect perceptual performance, such that object recognition is facilitated when an object is perceived within a scene that is typical for that particular object (e.g., a couch in the living room) compared to when an object is embedded in an untypical environment (e.g., a couch on the beach) (Bar 2004; Summerfield and Egner 2009; Zhao et al. 2013). A key question is how expectations from different processing levels relate to each other. The brain may form expectations simultaneously and independently on different processing levels and within different processing modules. One possibility is that expectations concerning one entity of visual information would be unaffected by expectations formed about other aspects of visual information.

Evidence for this assumption comes from a recent fMRI study by Stefanics et al. (2019). The authors tested whether the same physical stimulus produced distinct feature-specific prediction errors for the color and emotional expression of faces. Their results suggest that violations of different feature expectations are processed in different brain regions and do not interact when the features are unattended and task-irrelevant.

Alternatively, expectations from various processing levels may be combined to form a joint expectation when they are perceptually related, for instance, when they refer to the same object. Recent evidence in favor of the latter assumption comes from a study by Jiang, Summerfield and Egner (2016). The authors performed a behavioral experiment in which colored moving dots were presented. The dots could be either red or green and move upwards or downwards. An auditory cue indicated the upcoming color and movement direction with a validity of 75%, and participants were instructed to attend to either the color or motion of the stimuli presented. In particular, participants were asked to identify the color or the motion direction of the dots. Three competing hypotheses were tested. First, expectations about color and motion operate independently, and the violation of one feature expectation does not affect the other (“independence model”). Second, a prediction error for one feature spreads to the other due to an expectation at the object level (“reconciliation model”). Third, the conflict between expected and unexpected features will result in the perception that both features do not belong to the same object and produce segregated representations for each feature (“segregation model”). The results suggested that expectancy-related reaction time benefits did affect not only the attended but also the unattended feature, thereby supporting the assumptions of the reconciliation model that prediction errors referring to the same object are combined. Yet, at least in principle, prediction errors emerging within different visual dimensions may interact irrespective of whether or not they are bound to the same object. Testing this hypothesis requires an experimental variation assigning prediction errors from different dimensions to different objects. Accordingly, feature expectations were manipulated on the same or different objects in the current study. To prevent potential confounding effects originating from response-consistent perceptual and motor expectations, feature expectations induced in the present experiment were defined as task-irrelevant and were, hence, not related to any particular motor response.

We hypothesized that for combined object-level expectancies (i.e., for expectancies about features on the same object), the simultaneous violation of two feature expectations would result in an interaction of both prediction errors, which would reflect a mutual influence of both prediction error signals in that the joint prediction error signal is less or more than the sum of its parts. In contrast, the prediction errors for both features should be independent (i.e., additive) and not interact when the features are distributed to separate objects.

## Experiment 1

Experiment 1 was conducted to determine whether it is possible to manipulate feature expectations independently on two task-irrelevant dimensions when the features are separated on different objects. We presented two sinusoidal grating stimuli, where one feature (color) was manipulated on one grating and the other feature (orientation) on the other grating.

Expectations were manipulated by presenting specific feature configurations more frequently than others, assuming that the biased probabilities of the features of the target objects would be learned implicitly. This setup resulted in four experimental conditions: (1) color and orientation expected, (2) color expected and orientation unexpected, (3) color unexpected and orientation expected, and (4) color and orientation unexpected.

The participants’ task was focused on yet a further stimulus feature and they were asked to report whether the spatial frequencies of both target gratings were identical or different. Thereby, the participants’ task required them to keep track of and to respond to this third dimension (e.g., frequency), so that it was orthogonal to the expectations related to color and orientation.

We hypothesized that violations of feature expectations for color and orientation will affect behavior (response times) independently (i.e., additively), and will, hence, not interact even when both feature expectations are violated simultaneously.

## Materials and methods

### Participants

Sixteen participants (five women, mean age: 29.2 years, age range: 19—46, one left-handed) took part in Experiment 1. All participants had a normal or corrected-to-normal vision and no history of neurological or psychiatric disorders. Normal color vision in all participants was assessed by pseudo-isochromatic color plates (Velhagen and Broschmann 2003). Before the experiment, written informed consent was obtained following the Declaration of Helsinki. The study was approved by the ethics committee of the German Society of Psychology, and participants were remunerated for their time.

### Apparatus

Stimuli were presented on a 22-in. Samsung SyncMaster monitor (spatial resolution 1680 X 1050; refresh rate 120 Hz) at a distance of 60 cm. A chin and forehead rest preserved the distance. The presentation of stimuli and response recording were controlled using PsychoPy psychology software for Python (Peirce 2007, 2008). Participants were provided with button response pads (NAtA Technologies) for each hand and responded by pressing the corresponding button on the button response pad with the left and right index fingers.

### Stimuli and task

**Fig. 1:**
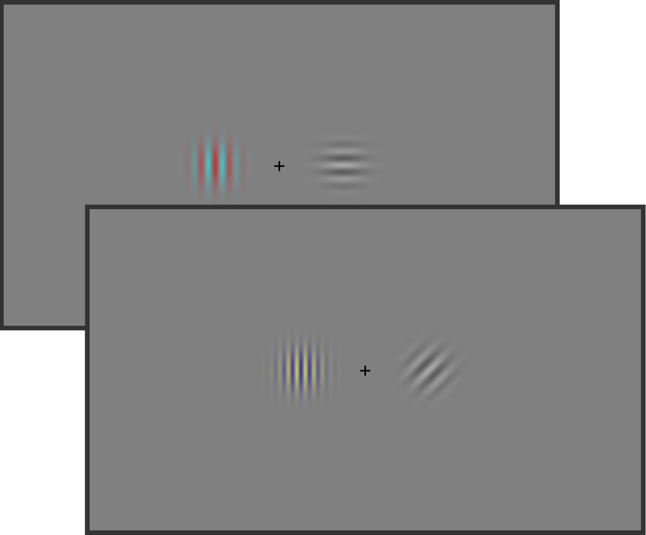
Stimulus examples of Experiment 1. The participants were asked to indicate by button presses whether the two gratings had the same or different spatial frequency. The probabilities of occurrence of the colors of one grating and the orientations of the other grating were manipulated to induce feature expectations

The visual stimuli consisted of two horizontally arranged target stimuli (see Fig. 1). A central black plus sign (0.57° × 0.57°) was placed in between serving as a fixation point. All stimuli were presented on a gray background. Participants were instructed to fixate the cross throughout the experiment.

The target stimuli were grating stimuli which consisted of a 4° × 4° sine wave grating windowed by a two-dimensional Gaussian envelope with a standard deviation of 0.66° with two possible spatial frequencies (low frequency: 1.5 cycles per degree and high frequency: 2.5 cycles per degree). All combinations of frequencies were presented randomly and with an equal probability (e.g., 50% same and 50% different).

Furthermore, one grating (color target) was always colored (either red/green or blue/yellow) and was oriented vertically (0°). The other grating (orientation target) was in grayscale, but could have two different orientations (45°, 90°). The side on which the color and the orientation targets were presented was held constant during the experiment (e.g., color was always left and orientation always right), but was counterbalanced across participants.

For the color target, one color combination (e.g., blue/yellow) was defined as the “expected color” and the other combination as “unexpected color”. Likewise, one orientation (e.g., 45°) was defined as the “expected orientation” and the other orientation as “unexpected orientation”. For both, color and orientation, the expected feature was presented on 87.5% of the trials.

A 2 × 2 factorial design with the factors Color Prediction Error (high, low) (*ColPE*) and Orientation Prediction Error (high, low) (*OriPE*) resulted in four experimental conditions: *ColPE_low/OriPE_low* (color expected and orientation expected), *ColPE_high/OriPE_low* (color unexpected and orientation expected), *ColPE_low/OriPE_high* (color expected and orientation unexpected), and *ColPE_high/OriPE_high* (both color and orientation unexpected).

The experiment consisted of 14 blocks, each comprising 64 trials, resulting in 896 trials. The experiment comprised 700 *ColPE_low/OriPE_low* trials (78.125%), 84 *ColPE_high/OriPE_low* trials (9.375%), 84 *ColPE_low/OriPE_high* trials (9.375%), and 28 *ColPE_high/OriPE_high* trials (3.125%).

Each trial started with the presentation of the two target stimuli until a response was given. An inter-trial interval, which randomly varied between 500 and 1000 ms, separated the trials.

Each block was followed by a break that could be terminated via button press.

The participants’ task was to indicate whether both target stimuli were identical or different concerning spatial frequency by pressing the corresponding response button with the left or right index fingers. The task was independent of the expectation manipulations of color and orientation, to avoid any confounding effects of response preparation to the features. Participants were asked to respond as fast and accurately as possible. An erroneous response produced the message “Fehler” (i.e., the German word for “error”) on the screen for 750 ms.

To familiarize the participants with the task, participants performed a training session of 128 trials before they started with the experiment. During the training, all trials were *ColPE_low/OriPE_low* trials. This was intended to let the participants form expectations about the most likely color and orientation of the target stimuli.

All participants were informed that the color and the orientation could change during the main experiment. Furthermore, they were told that color or orientation changes are irrelevant to their task.

### Analysis

The free statistical software R (R Foundation for Statistical Computing, Vienna, Austria; https://www.r-project.org) was used for behavioral data analysis. For each participant, mean RTs and error rates were calculated. Error trials, and trials following errors and trials with RTs differing more than two standard deviations from the mean were excluded from RT analysis.

Repeated-measures ANOVAs for the RTs and error rates were conducted with the within-subject factors *ColPE* (high, low) and *OriPE* (high, low). The reported mean values for expected and unexpected color and orientation were calculated by collapsing all trials with the specific feature being expected or unexpected (e.g., the mean values for the expected color reflect the mean of all *ColPE_low/OriPE_low* and *ColPE_low/OriPE_high* trials).

## Results

The overall amount of incorrect responses was very low with on average 2.19% (± 0.34 SEM) errors. The ANOVA of the error rates yielded a significant main effect of *OriPE *(*F*(1,15) = 5.025, *p*  <  0.05, ${\eta }_{P}^{2}$ = 0.186) with lower error rates for expected orientations (2.04%) compared to unexpected orientations (3.29%). Neither the main effect *ColPE*, with 2.02% errors for expected colors versus 3.40% errors for unexpected colors, was significant (*F*(1,15) = 3.437, *p* = 0.0835, ${\eta }_{P}^{2}$ = 0.251), nor was the interaction between *ColPE* and *OriPE* (F(1,15) = 1.453, *p* = 0.247, ${\eta }_{P}^{2}$ = 0.088).

**Fig. 2:**
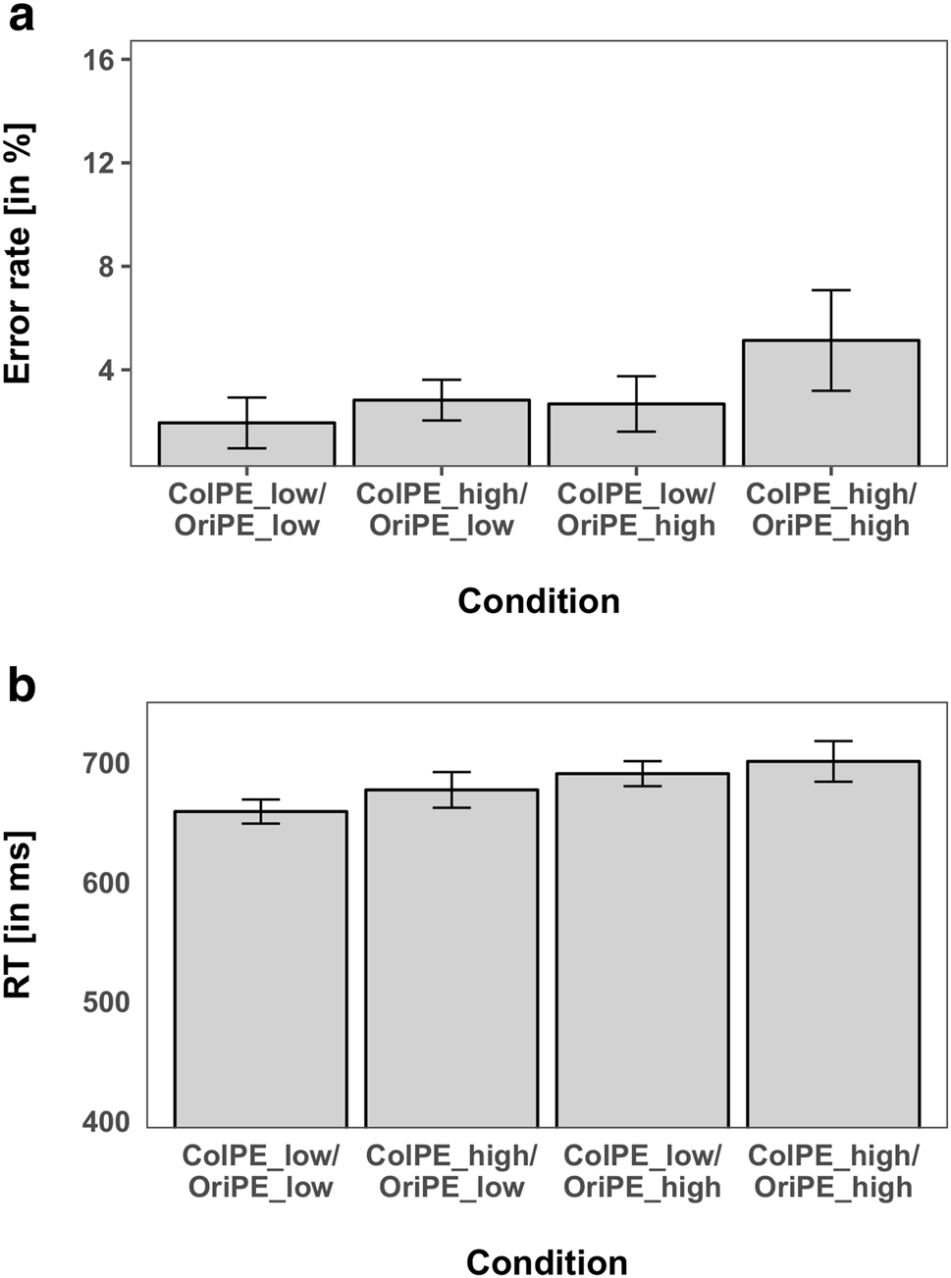
Performance measures of each combination of color and orientation manipulations of Experiment 1. **a** Error rates. **b** Reaction times. Error bars reflect the 95% confidence intervals

The ANOVA of the mean RTs revealed a significant main effect for *ColPE* (*F*(1,15) = 13.31, *p* < 0.05, ${\eta }_{P}^{2}$ = 0.470) with 662 ms for expected colors versus 682 ms for unexpected colors, reflecting RT costs for the unexpected colors. Moreover, we observed a significant main effect for *OriPE* (*F*(1,15) = 5.735, *p* < 0.05, ${\eta }_{P}^{2}$ = 0.277), with 660 ms for expected orientations versus 692 ms for unexpected orientations, reflecting a cost for unexpected orientation. Thus, both unexpected colors and unexpected orientations resulted in significantly higher RTs compared to the expected features, although both features were task-irrelevant and orthogonal to the actual task. The interaction of *ColPE* × *OriPE* was not significant (*F*(1,15) = 0.401, *p* = 0.536, ${\eta }_{P}^{2}$ = 0.026), providing no evidence that prediction errors for both features influenced each other. The mean RTs and error rates are shown in Fig. 2.

## Discussion

Experiment 1 investigated whether two simultaneous feature expectations can be manipulated independently. We hypothesized that feature expectation would be processed independently when the features are separated on different objects. Consistent with the hypothesis, the experiment provides evidence that separated feature expectations are processed independently.

When color and orientation features were expected due to their higher probability of occurrence, participants responded faster than in trials with one unexpected feature. Response times increased further in trials with two unexpected features compared to one unexpected. However, the RTs for two unexpected features increased additively rather than interactively, indicating independent effects for both features. These effects were observed despite color and orientation being irrelevant for the task of the subjects.

To investigate whether multiple simultaneous feature expectations of the same object result in a combined object-level expectation, we conducted a second experiment.

## Experiment 2

Experiment 2 was designed to determine whether concurrent color and orientation expectancies combine interactively when both features are bound to the same object. In Experiment 1, the color and orientation features were distributed to separate objects, which, consequently, did not result in an interaction of both types of prediction errors. Experiment 2 followed the procedure of Experiment 1, except that in Experiment 2, both color and orientation expectations were manipulated on both gratings simultaneously. We hypothesized that simultaneous violations of both feature expectations for color and orientation within the same object would result in an interaction, indicating combined object-level expectancies.

## Materials and methods

### Participants

Sixteen participants (eight women, mean age: 29.69 years, age range: 20–45, two left-handed) took part in Experiment 2. Seven of them already participated in Experiment 1. All participants had a normal or corrected-to-normal vision and no history of neurological or psychiatric disorders. Normal color vision in all participants was assessed by pseudo isochromatic color plates (Velhagen and Broschmann 2003). Before the experiment, written informed consent was obtained following the Declaration of Helsinki. The study was approved by the ethics committee of the German Society of Psychology, and participants were remunerated for their time.

### Stimuli, design, and procedure

**Fig. 3:**
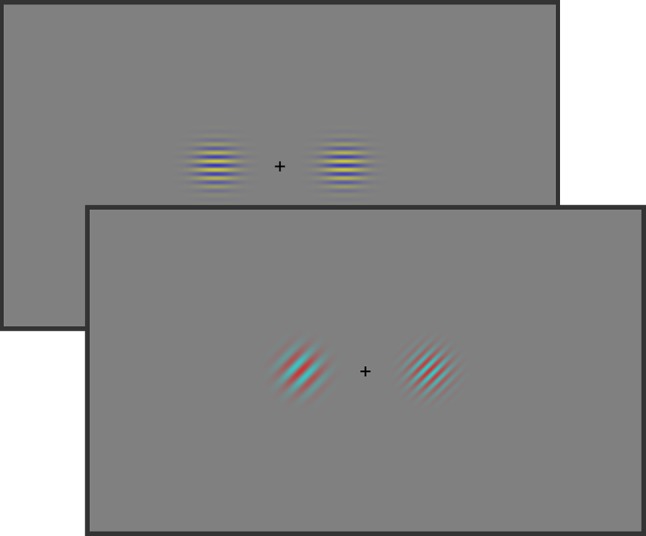
Stimulus examples of Experiment 2. As in Experiment 1, participants were asked to respond to the spatial frequency of the two gratings, which could be the same or different. The probabilities of occurrence of both color and orientation of the identical gratings were manipulated to induce feature expectations

In general, procedures were similar to Experiment 1. Again, the participants’ task was to indicate whether the spatial frequencies of two gratings stimuli were identical or different. In contrast to Experiment 1, however, both color and orientation expectation were manipulated on the two gratings simultaneously (see Fig. 3). The probabilities for the expected and unexpected features were identical to those in Experiment 1.

Like in Experiment 1, participants performed a training session of 128 trials with 100% expected features before they started with the experiment.

### Analysis

In general, the analysis of Experiment 2 was identical to Experiment 1. In an additional analysis, we compared data across Experiment 1 and Experiment 2, to test whether simultaneous prediction errors of color and orientation on the same objects resulted in a combined expectancy effect. Therefore, for both experiments, the mutual interaction of prediction errors in the different dimensions was estimated by calculating an interaction score (ISC) that contrasts the effects of simultaneous high prediction errors in both dimensions with high prediction errors in only one dimension. Trials with only a prediction error in one dimension (*ColPE_low*/*OriPE_high and ColPE_high*/*OriPE_low)* were subtracted from the sum of trials with consistent prediction errors in both dimensions *(ColPE_high/OriPE_high and ColPE_low/OriPE_low).* In particular, the calculation can be expressed by the interaction score ISC = *(ColPE_high*/*OriPE_high* + *ColPE_low*/*OriPE_low)—(ColPE_high*/*OriPE_low* + *ColPE_low*/*OriPE_high).* Please note that in both terms, the same number of high and low prediction errors are involved, the only difference being that in one term prediction errors occurred simultaneously. In case there is absolutely no interaction (i.e., when the effects of a prediction error in one dimension is independent of a prediction error in another visual dimension), the ISC would be expected to be 0. Otherwise, if prediction errors in both dimensions jointly generate a higher prediction error, the ISC value will be positive. ISC values smaller than zero would be indicative of reduced prediction errors when both belong to the same object.

This calculation was conducted for RTs as well as for error rates. The resulting scores from both experiments were then compared using a *t* test for partially depending samples (Derrick et al. 2017).

## Results

Similar to Experiment 1, the mean error rate was very low with an average of 3.28% (± 0.38 SEM).

The ANOVA of the error rates yielded a significant main effect for *ColPE* (*F*(1,15) = 9.785, *p* < 0.05, ${\eta }_{P}^{2}$ = 0.395) with lower error rates for expected colors (2.97%) compared to unexpected colors (5.47%) and a significant main effect for *OriPE* (*F*(1,15) = 11.65, *p* < 0.05, ${\eta }_{P}^{2}$ = 0.437) with lower error rates for expected orientations (2.95%) compared to unexpected orientations (5.58%). The interaction was not significant (*F*(1,15) = 0.904, *p* = 0.357, ${\eta }_{P}^{2}$ = 0.057).

**Fig. 4:**
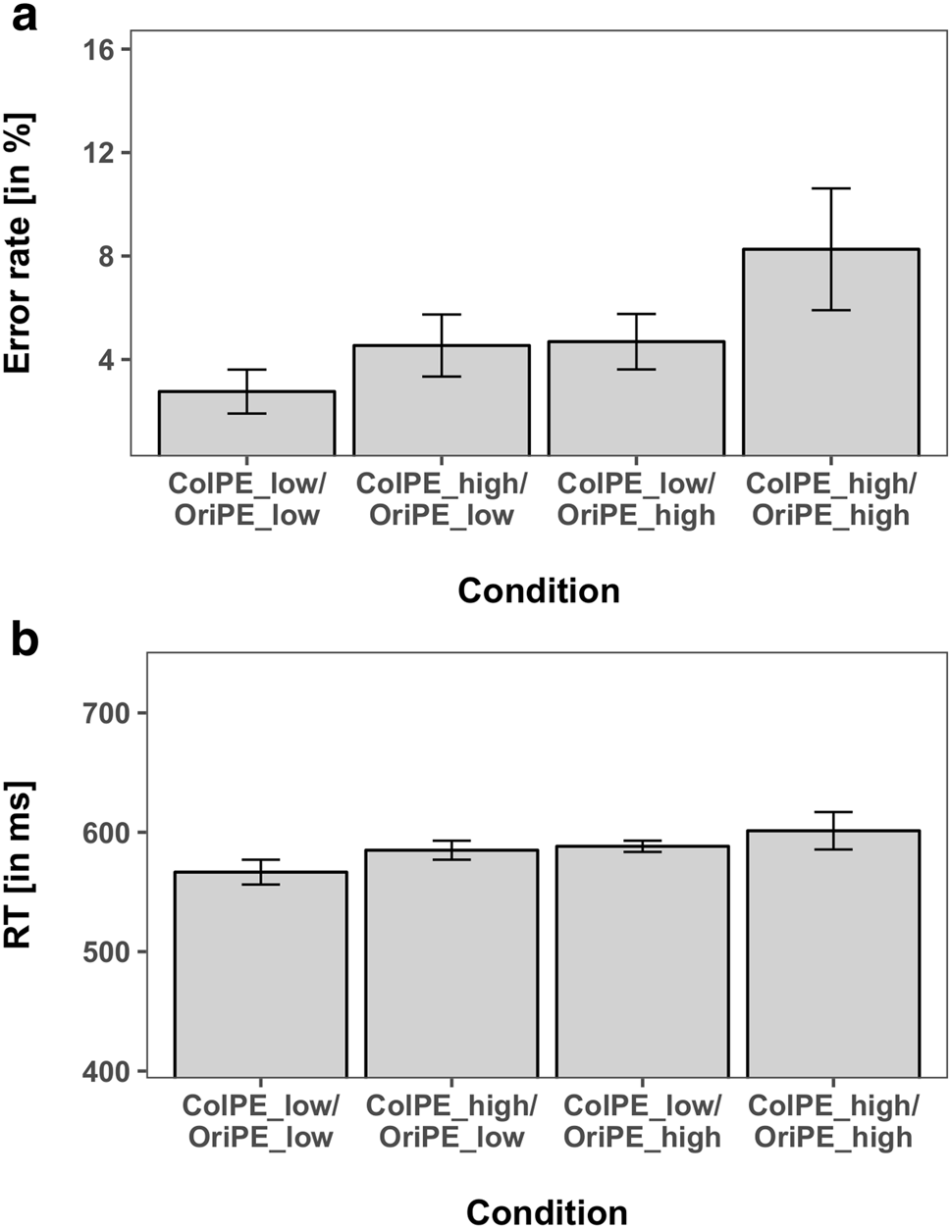
Performance measures of the combination of color and orientation manipulations of Experiment 2. **a** Error rates. **b** Reaction times. Error bars reflect the 95% confidence intervals

Again, the ANOVA of the mean RTs revealed a significant main effect for *ColPE *(*F*(1,15) = 8.035, *p* < 0.05,${\eta }_{P}^{2}$ = 0.349), with 569 ms for expected colors versus 591 ms for unexpected colors, and a significant main effect for *OriPE *(*F*(1,15) = 5.778, *p* < 0.05, ${\eta }_{P}^{2}$ = 0.278), with 569 ms for expected orientations versus 593 ms for unexpected orientations, reflecting the cost for unexpected features. Thus, both unexpected color and unexpected orientation stimuli resulted in significantly higher RTs compared to the more frequently presented standard targets. The interaction of *ColPE* × *OriPE* was not significant (F(1,15) = 0.329, p = 0.575, ${\eta }_{P}^{2}$ = 0.021), providing no evidence for a mutual influence of prediction errors for both features. Both feature prediction errors were hence again processed independently, even when they were part of the same object. The mean RTs and error rates are shown in Fig. 4.

A joint analysis of the combined data of Experiment 1 and Experiment 2 revealed no significant difference concerning the interaction score ISC of error rates between experiments (*t*(19.5) =  − 0.12, p = 0.909), with an ISC of 1.57 in Experiment 1 and 1.79 in Experiment 2. Similarly, the comparison of the ISC related to RT costs revealed no significant difference between experiments (*t*(19.5) =  − 0.15, *p* = 0.879), with an ISC of -8 in Experiment 1 and -5 in Experiment 2.

## Discussion

Experiment 2 was designed to determine whether concurrent color and orientation expectancies are combined interactively when both features are bound to the same object. We hypothesized that violations of both feature expectations simultaneously would result in mutual influence and hence in an interaction.

Consistent with Experiment 1, the RT costs associated with one feature being unexpected were also evident in Experiment 2 (as evidenced by the significant main effects for *ColPE* and *OriPE*). Contrary to our hypothesis, the results of Experiment 2 did not show an interaction between *ColPE* and *OriPE*, providing no evidence for a mutual influence of both types of prediction errors. This finding was confirmed by a direct comparison of the interaction pattern, as reflected in ISC determined in Experiment 1 and Experiment 2. In Experiment 1, the feature expectations for color and orientation were separated onto two different objects while the same feature expectations were combined and manipulated on the same objects simultaneously in Experiment 2. We hypothesized that combined object-level expectancies for color and orientation should be reflected in different ISCs related to the RT costs and error rates between experiments. However, this comparison revealed no significant difference and, again, did not provide any evidence for combined object-level expectancy effects.

These findings do not support the idea of combined feature expectancies on an object level, at least when expectancies are formed implicitly and when they relate to visual dimensions that are currently irrelevant for an ongoing task and are hence unattended. Attention has previously been suggested to play an essential role in feature binding. According to Treisman’s Feature Integration Theory (FIT), basic visual features are first processed independently on a preattentive level before attentional binding combines them into a single-object representation (Treisman and Gelade 1980). Following this argumentation, one may expect that without attentional binding, not only feature representations per se, but also related expectancies are coded separately. Conversely, when these features are attended and are hence integrated to whole object representations via attentional binding, then expectancies may also be formed by combined feature information. To investigate whether the lack of interaction was indeed due to the lack of attentive processing of the implicitly learned target features, a third experiment was conducted.

## Experiment 3

The results of Experiment 2 indicated that multiple feature expectations for task-irrelevant features influence behavior additively rather than interactively, even when the features belong to the same object. This result seems to be in line with the findings of Stefanics et al. (2019) who did not find an interaction of prediction errors of different features, when the features were unattended. Thus the absence of an interaction may be accounted for by a lack of attention assigned to the different features and hence by a lack of object binding in the current experiments. Therefore, Experiment 3 was designed to test whether the absence of an interaction between feature expectations was due to a lack of object binding. To promote object feature binding, we increased the subjects’ need to attend to the gratings’ color and orientation explicitly. Instead of manipulating feature probabilities of upcoming targets by their frequency of occurrence, we induced expectations of the upcoming feature configuration explicitly on a trial-by-trial basis using verbal cues.

Furthermore, we added a secondary task to the experiment, where participants had to estimate the percentage of cue validity after each experimental block. This approach allowed increasing the feature relevance without an association between the main task and a specific motor response.

## Materials and methods

### Participants

Sixteen participants (six female, mean age: 30.7 years, age range: 21–46, two left-handed) took part in Experiment 3. Seven of them had participated in the previous experiments, and one had participated in Experiment 2. All participants had a normal or corrected-to-normal vision and no history of neurological or psychiatric disorders. Normal color vision in all participants was assessed by pseudo-isochromatic color plates (Velhagen and Broschmann 2003). Before the experiment, written informed consent was obtained following the Declaration of Helsinki. The study was approved by the ethics committee of the German Society of Psychology, and participants were remunerated for their time.

### Stimuli, design, and procedure

In general, procedures were very similar to Experiment 2. The participants’ task was again to indicate whether the spatial frequencies of two gratings were identical or different, and the occurrence of different colors and orientations was manipulated for both gratings simultaneously. However, in contrast to Experiment 1 and 2, the probability for each color and orientation was identical, e.g., 50% red/green and 50% blue/yellow, as well as 50% horizontal and 50% tilted orientation. Each trial started with a verbal cue, indicating the most likely color and orientation for the next trial for 583 ms. Explicit cues comprised the written words of the likely upcoming color [“gelb” or “rot” (i.e., “yellow”, “red”)] and the likely upcoming orientation (“horizontal” or “diagonal”). To keep the information provided by the cue minimal, only one color of the color combinations was cued (e.g., “yellow” instead of “blue/yellow”). The cue validity was equivalent to the feature probabilities of Experiments 1 and 2. Hence, the cue was valid in 78.125% of the trials for both features; in 9.375%, the color information was invalid; likewise in 9.375% of the trials, the cue was invalid regarding the orientation, and in 3.125% of the trials, both the color and the orientation information were invalid. The number of valid and invalid cues was the same for all blocks. The cue and target stimuli were separated by a cue–target interval, which randomly varied between 250 and 500 ms.

Additionally, to increase the relevance of the color and orientation features, a secondary task was added to the experiment. Participants were asked to report their belief about the cue validity at the end of each block, using a rating scale ranging from 0 to 100% (in 5% steps). Participants were not aware that the overall validity was the same for all the blocks.

As in the previous experiments, participants performed a training session of 128 trials with 100% expected features before the experiment.

### Analysis

In general, the analysis of Experiment 3 was identical to Experiment 2. Again, the results of Experiment 3 were compared with the previous experiment’s results. Additionally, to test whether explicitly formed expectations affect behavior differently than implicit formed expectations, RT costs and error rates for ColPE [(*ColPE_high/OriPE_low)–(ColPE_low/OriPE_low)]* and OriPE [(*ColPE_low/OriPE_high)–(ColPE_low/OriPE_low)]* were compared between Experiment 2 and Experiment 3.

To test whether participants perceived the verbal cues as valid, two one-sample *t* tests were conducted. First, to test whether participants assigned any predictive value to the verbal cues at all, we tested whether the estimated cue validities were better than chance level (i.e., significantly different from 50%). Second, to test whether participants were able to infer the actual validity, estimated cue validity values were compared against the true cue validity of 78.125%.

## Results

Participants estimated cue validity for all blocks on average at 67.46% (± 2.33% SEM). A one-sample *t *test indicated that this value was significantly different from chance level (50%) (*t*(15) = 7.482, *p* < 0.05). However, cue validity as perceived by the participants was also lower than the actual cue validity of 78.125% as indicated by a second one-sample *t* test (*t*(15) =  − 4.573, *p* < 0.05).

Similar to the previous experiments, the mean error rate was low, with an average of 4.65% (± 0.65 SEM). The ANOVA of the error rates showed neither significant main effects of *ColPE* (*F*(1,15) = 4.028, *p *= 0.0631, ${\eta }_{P}^{2}$ = 0.211), with 4.23% for expected colors versus 7.59% for unexpected colors, nor a significant main effect of *OriPE *(*F*(1,15) = 1.219, *p* = 0.287, ${\eta }_{P}^{2}$ = 0.075), with 4.43% for expected orientations versus 6.14% for unexpected orientations. The interaction between the two factors was not significant (*F*(1,15) = 0.486, *p* = 0.496, ${\eta }_{P}^{2}$ = 0.314).

**Fig. 5:**
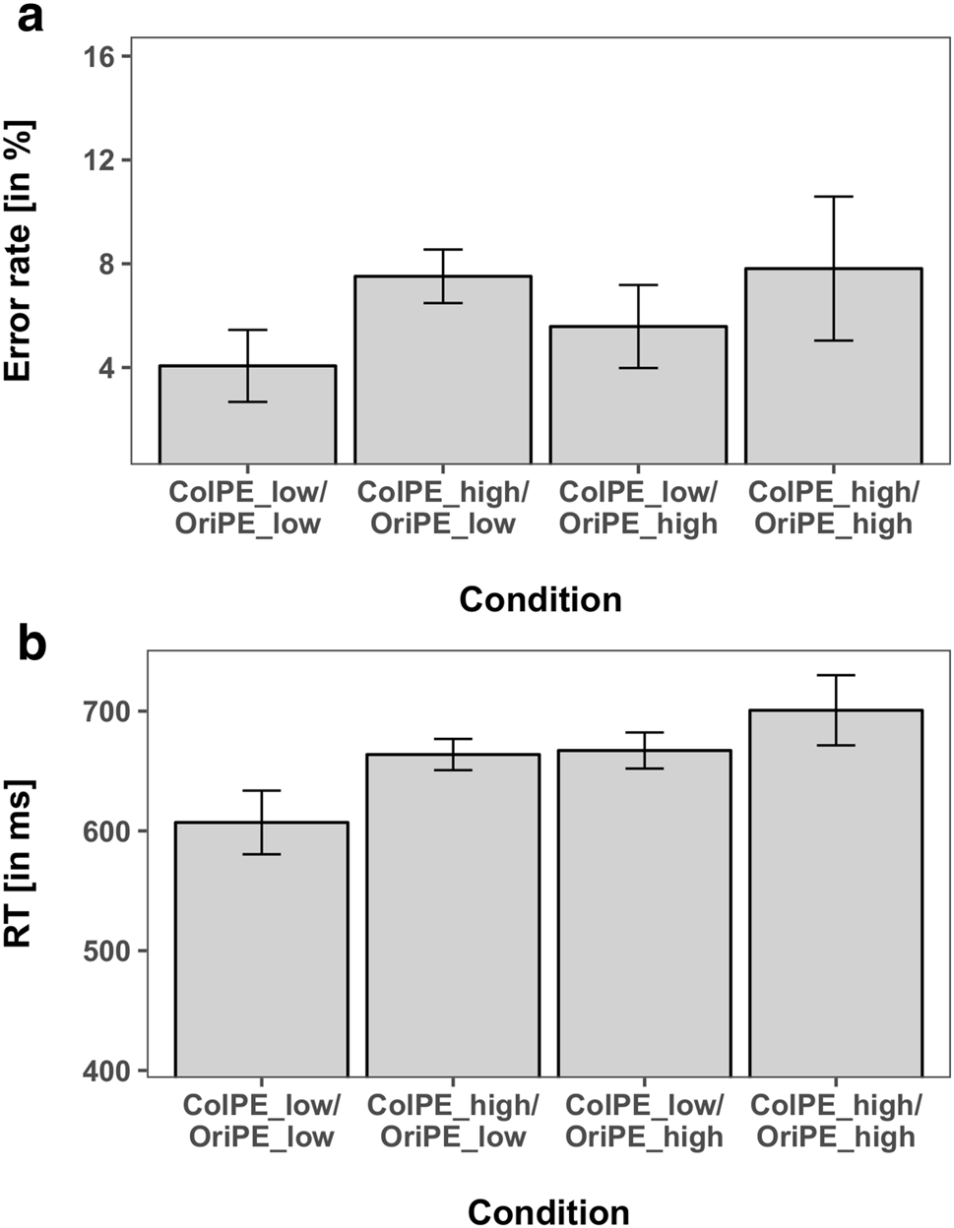
Performance measures of the combination of color and orientation manipulations of Experiment 3. **a** Error rates. **b** Reaction times. Error bars reflect the 95% confidence intervals

As in the previous experiments, the ANOVA of the mean RTs revealed significant main effects for *ColPE *(*F*(1,15) = 14.31, *p* < 0.05, ${\eta }_{P}^{2}$ = 0.488), with 612 ms for expected colors versus 676 ms for unexpected colors, and *OriPE *(*F*(1,15) = 10.08, *p* < 0.05, ${\eta }_{P}^{2}$ = 0.402), with 613 ms for expected orientations versus 676 ms for unexpected orientations, reflecting the cost for unexpected stimuli. Similar to the previous experiments, both unexpected color and orientation stimuli resulted in significantly higher RTs compared to the standard targets. Likewise, the interaction of *ColPE* × *OriPE* was again not significant (*F*(1,15) = 0.993, *p* = 0.335, ${\eta }_{P}^{2}$ = 0.062). The mean RTs and error rates are shown in Fig. 5.

A joint analysis of the combined data from Experiment 2 and Experiment 3 revealed no significant difference in error rates for color expectation between experiments [Experiment 2: 2.76% (expected) vs. 4.54% (unexpected); Experiment 3: 4.06% (expected) vs. 7.51% (unexpected); *t*(18.5) =  − 1.65, *p* = 0.116] However, there was a significant difference in error rates for orientation expectation between experiments [Experiment 2: 2.76% (expected) vs. 4.69% (unexpected); Experiment 3: 4.06% (expected) vs. 5.58% (unexpected); *t*(18.5) = 0.49, *p* < 0.05]. Additionally, there was no significant difference in error rates between the experiments for simultaneous prediction errors of color and orientation (*t*(18.5) = 1.20, *p* = 0.244), with regard to the ISC of error rates between experiments, with an ISC of 1.79 in Experiment 2 and 1.22 in Experiment 3.

Comparing RTs from Experiment 2 with those from Experiment 3 revealed a significant difference in RT costs for unexpected colors between the experiments (*t*(18.5) =  − 2.39, *p* < 0.05), with unexpected colors resulting in an RT increase of 18.36 ms in Experiment 2 versus an increase of 56.72 ms in Experiment 3. The comparison of RTs for the orientation expectation between experiments was non-significant (*t*(18.5) =  − 1.87, *p* = 0.077), with unexpected orientation resulting in 21.65 ms higher RTs in Experiment 2 versus an increase of 60.08 ms in Experiment 3. The comparison of RT costs for simultaneous prediction errors of color and orientation between experiments was non-significant concerning the ISC-related RT costs (*t*(18.5) = 0.92, *p* = 0.369), with an ISC of -5 in Experiment 2 and  − 23 in Experiment 3.

## Discussion

Experiment 3 was conducted to examine whether the lack of an interaction between the feature expectations observed in Experiment 2 was due to the implicitly learned probabilities of the task-irrelevant features that may have prevented object feature binding. To increase the demands for participants to attend to the color and orientation features, we manipulated feature expectations in Experiment 3 explicitly on a trial-by-trial basis through verbal cues. Furthermore, to make the features relevant, a secondary task was added to the experiment. Participants were asked to report their estimate about the cue validity after every experimental block.

The estimated validity differed from the true cue validity of 78.125%, indicating that participants underestimated cue validity. However, participants noticed the predictive value of the cue as indicated by validity estimates different from chance level. Not only that they perceived cues as valid, they also used the cue information to prepare for upcoming stimulus configurations, as can be seen from the reaction time pattern. In particular, participants responded faster in trials involving validly cued features rather than invalidly cued features, reflecting the RT costs for invalidly cued and hence unexpected features. This pattern is consistent with the reaction time pattern observed in the previous experiments where participants responded faster in trials with expected rather than unexpected features. Hence, the findings from Experiment 3 indicate that the verbal cues successfully manipulated feature expectations. Additionally, as in the previous experiments, RTs for two unexpected features increased additively rather than interactively, providing no evidence for joint expectations regarding both features. This result suggests that the effects observed in Experiment 2 were not specific to the implicit nature of feature expectancy.

To test whether the explicitly formed feature expectations affected the processing of prediction error concerning these expectations differently than the implicitly learned expectations of Experiment 2, we compared the results of both experiments. The analysis revealed significantly higher RT costs for unexpected colors in Experiment 3 than in Experiment 2, but not for unexpected orientations. Furthermore, unexpected orientations but not unexpected colors resulted in significantly higher error rates in Experiment 2 than in Experiment 3. This finding shows that explicitly formed expectations affect the behavior differently than implicitly formed expectations. This effect was not consistent across both visual dimensions, and the behavioral pattern indicated an asymmetry about explicitly formed expectations, such that the effects of prediction errors based on explicitly formed expectations increased for color and decreased for orientation. This result might either reflect an attentional bias towards color in Experiment 3, rendering prediction errors in the color domain more relevant. Alternatively, predictions concerning orientation may be formed more implicitly than explicitly.

## Experiment 4

The previous experiments indicated that expectations regarding different visual features affect behavior additively rather than interactively. This was true even when these features belonged to the same object. Moreover, this effect was likewise observed for implicit and explicit expectations. The task performed in these experiments required participants to simultaneously attend to two objects, thereby reducing the attentional resources available for each single object. Since attention has been suggested to be a necessary prerequisite for feature binding (Treisman and Gelade 1980), divided attention might possibly decrease the degree of feature binding and accordingly potential interactions between feature expectations. This interpretation seems consistent with findings from previous studies. Evidence for a separate coding of feature expectations has been reported in a study using multiple non-foveated stimuli (Stefanics et al. 2019), whereas a study using a single central stimulus provided evidence in favor of an interaction between feature predictions (Jiang et al. 2016). In Experiment 4, we, therefore, tested whether an interaction between feature expectations would be observed when feature binding was maximized by manipulating feature expectations of a single central grating.

## Materials and methods

### Participants

Sixteen participants (seven female, mean age: 32.6 years, age range: 23–47, two left-handed) took part in Experiment 4. All participants had a normal or corrected-to-normal vision and no history of neurological or psychiatric disorders. Normal color vision in all participants was assessed by pseudo-isochromatic color plates (Velhagen and Broschmann 2003). Before the experiment, written informed consent was obtained following the Declaration of Helsinki. The study was approved by the ethics committee of the German Society of Psychology, and participants were remunerated for their time.

### Stimuli, design, and procedure

**Fig. 6:**
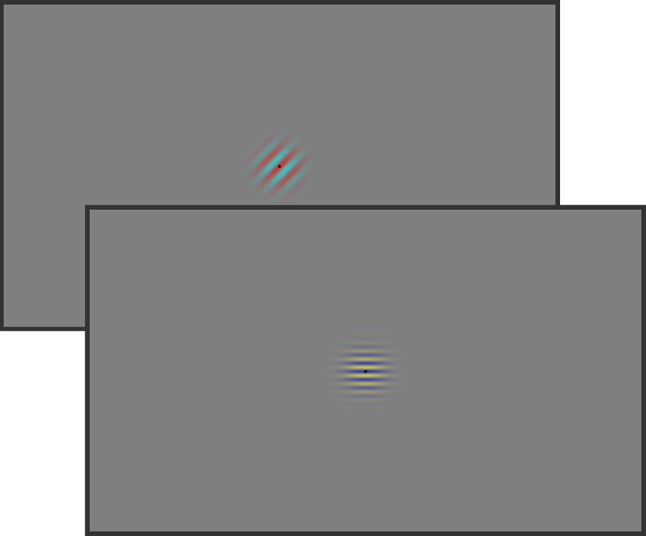
Stimulus examples of Experiment 4. Participants were asked to respond to the spatial frequency of the grating, which could be high or low. The probabilities of occurrence of both color and orientation of the single grating were manipulated to induce feature expectations

In general, procedures were similar to Experiment 2. However, in contrast to Experiment 2, only a single central grating with a fixation point was presented in Experiment 4 (Fig. 6). Moreover, the task this time was to indicate whether the spatial frequency of the grating was either high or low. The probabilities of the different color and orientation features were identical to those in Experiment 1 and Experiment 2. As in the previous experiments, participants performed a training session of 128 trials with 100% expected features before the experiment.

### Analysis

The analysis of Experiment 4 was identical to Experiment 1.

## Results

Similar to the previous experiments, the mean error rate was low, with an average of 5.33% (± 0.65 SEM).

The ANOVA of the error rates yielded a significant main effect for *ColPE* (*F*(1,15) = 5.811, *p* < 0.05, ${\eta }_{P}^{2}$ = 0.279), with lower error rates for expected colors (4.95%) compared to unexpected colors (8.04%), and a significant main effect for *OriPE* (*F*(1,15) = 8.356, *p* < 0.05, ${\eta }_{P}^{2}$ = 0.358) with lower error rates for expected orientations (4.91%) compared to unexpected orientations (8.31%). The interaction was not significant (*F*(1,15) = 0.908, *p* = 0.356, ${\eta }_{P}^{2}$ = 0.057).

**Fig. 7:**
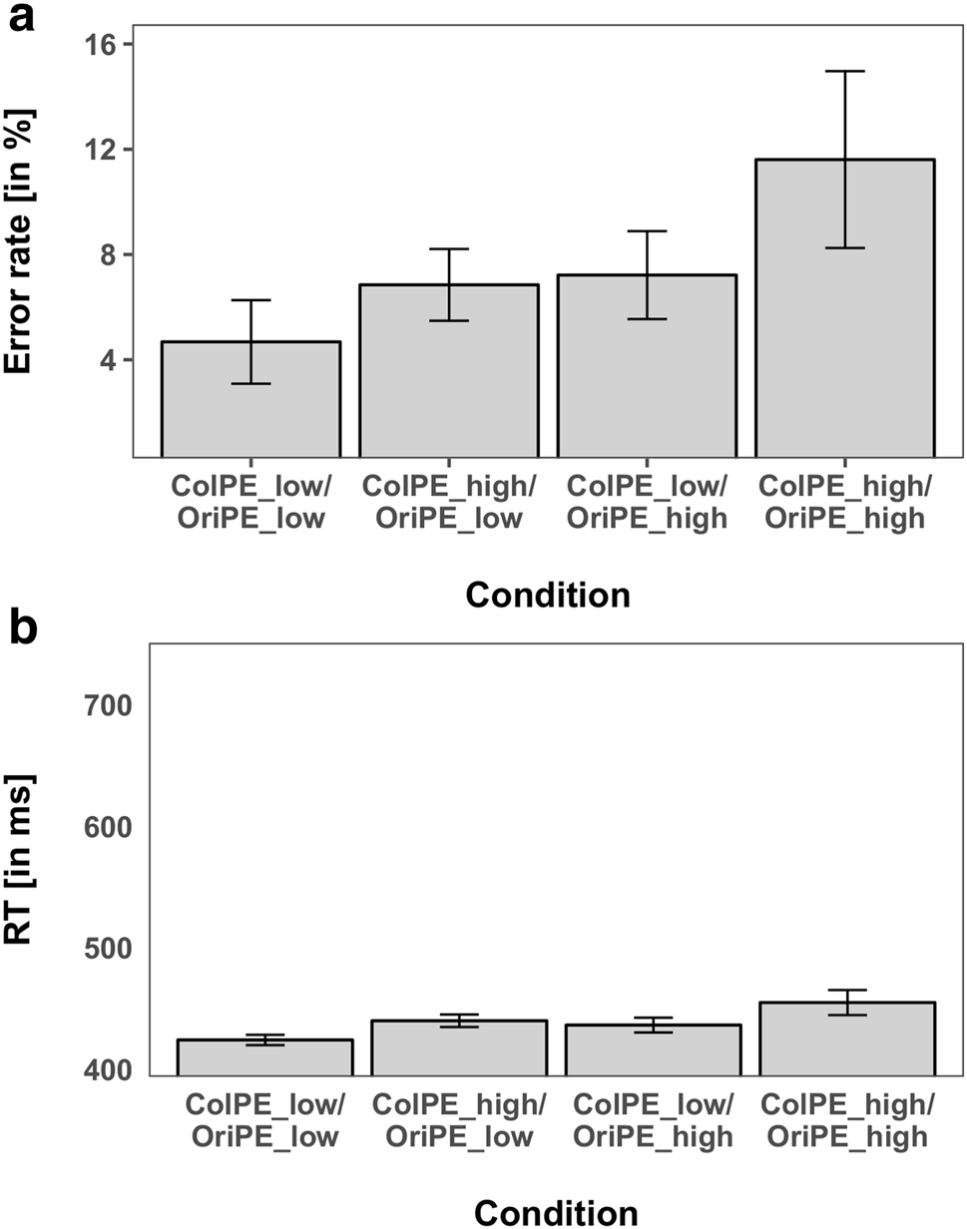
Performance measures of the combination of color and orientation manipulations of Experiment 4. **a** Error rates. **b** Reaction times. Error bars reflect the 95% confidence intervals

Again, the ANOVA of the mean RTs revealed a significant main effect for *ColPE *(*F*(1,15) = 16.559, *p* < 0.05, ${\eta }_{P}^{2}$ = 0.525), with 427 ms for expected colors versus 444 ms for unexpected colors, and a significant main effect for *OriPE *(*F*(1,15) = 15.17, *p* < 0.05, ${\eta }_{P}^{2}$ = 0.503), with 427 ms for expected orientations versus 442 ms for unexpected orientations, reflecting the cost for unexpected features. Thus, both unexpected color and unexpected orientation stimuli resulted in significantly higher RTs compared to the more frequently presented standard targets. The interaction of *ColPE* × *OriPE* was not significant (*F*(1,15) = 0.101, *p* = 0.75, ${\eta }_{P}^{2}$ = 0.007), providing no evidence for a mutual influence of prediction errors for both features. Hence, both feature prediction errors were again processed independently, even when they were part of the same object. The mean RTs and error rates are shown in Fig. 7.

## Discussion

Experiment 4 was conducted to examine whether the lack of an interaction between feature expectations observed in Experiments 1–3 can be explained by reduced object feature binding due to divided attention across multiple objects. Therefore, in Experiment 4, only one single central stimulus was presented, which was used to manipulate color and orientation expectations. Consistent with the previous experiments, the RT costs associated with one feature being unexpected were also evident in Experiment 4. Furthermore, as in the previous experiments and despite a higher degree of feature object binding, RTs for two unexpected features increased additively rather than interactively providing no evidence for joint expectations regarding both features. Accordingly, the lack of an interaction between prediction errors in different visual features in the previous experiments cannot be accounted for by a need to divide the attentional focus.

## General discussion

This study investigated whether expectations regarding multiple visual features of different dimensions (color and orientation) are formed independently when they refer to the same object, or whether feature expectations are combined to a joint object-level expectation. A previous study by Jiang et al. (2016) reported behavioral and functional imaging evidence supporting the latter assumption. In particular, they suggested that a prediction error about one feature spreads across other object features and marks the entire object as “unexpected”. In the current study, four behavioral experiments were performed to systematically manipulate the distribution of feature expectations to the same or different objects and, hence, to extend the findings of Jiang et al. (2016).

The newly developed paradigm allowed inducing prediction errors in different visual dimensions for the same or different objects. Furthermore, the paradigm allowed investigating prediction errors emerging within two task-irrelevant feature dimensions and hence avoided any potential confounds with response-related prediction errors in the motor domain. In a first experiment, prediction errors were induced in the color and orientation dimension, and each type of prediction error was confined to separate objects. Both unexpected color and unexpected orientation increased RTs, indicating that the paradigm successfully elicited prediction error signals of task-irrelevant feature dimensions and that these predictive error signals, although task-irrelevant, altered behavior in an ongoing task. Prediction errors in both visual dimensions were comparable in magnitude and showed no signs of mutual influence as indicated by an additive rather than an interactive RT pattern. Therefore, they appear to be calculated separately when the features are distributed between separate objects.

We then tested whether this pattern changed when prediction errors in different dimensions co-occurred within the same object. Again, prediction errors in both dimensions reliably induced slower RTs when unexpected features were presented. As in the previous experiment and contrary to our initial hypothesis, RTs showed an additive rather than an interactive pattern. Accordingly, even when prediction errors were induced by features belonging to the same object, we could not find evidence for mutual influence and interference between dimensions. One possible explanation for this finding is that feature expectations in the present experiments were generated implicitly and may, hence, be formed before object binding occurs, a process that has critically been associated with focused attention (Treisman and Gelade 1980). To test this, an additional experiment was conducted where feature expectations were induced by verbal cues, hence increasing top-down aspects of feature expectations. Despite a more explicit representation of features expectations, no evidence for combined feature expectations was observed and the RT patterns found in Experiments 1 and 2 were replicated. These patterns persisted even when a single-object version of our task was used. This variant was introduced to increase the amount of attention allocated to a single target object and thereby the degree of object feature binding. No evidence for a mutual interaction of feature expectations was found, although visual features were part of an integrated object representation.

In sum, this series of experiments yielded no evidence for a mutual influence of prediction errors in different dimensions. This finding was independent of whether feature expectations were formed implicitly or explicitly and whether or not attention was fully engaged at a single target object.

Our results are thus in line with results reported by Stefanics et al. (2019) in the sense that task-irrelevant feature predictions do not interact. However, the finding of an independent coding of feature predictions seems at contrast with a study by Jiang et al. (2016) which reported a cross-blending of prediction errors. A crucial difference between the study by Stefanics et al. (2019) and our study on one hand and the study by Jiang et al. (2016) on the other hand is related to a feature’s task relevance. Evidence for an interaction of prediction errors was observed only in the study by Jiang et al. (2016) where prediction errors in a task-irrelevant dimension affected predictions in a task-relevant dimension. In the present study, prediction errors were manipulated in two task-irrelevant dimensions and no interactions were found between the two. However, the present study involved spatial frequency as an additional task-relevant dimension. Expectations with regard to this dimension were held neutral, with both feature values being equally likely across the experiment. Prediction errors in the task-irrelevant dimensions interfered with spatial frequency judgements in the task-relevant dimension and increased reaction times. A possible explanation for this interference is that prediction errors emerging in the task-irrelevant dimension alter prediction errors in the task-relevant dimension, rendering the neutral but task-relevant feature unexpected. In this case, the results could be taken as support for the view that combined expectancies critically depend on task or response relevancy. However, it is unclear whether interference of task-irrelevant and task-relevant features is in fact based on a combination of prediction errors. Inconsistent prediction errors may also result in more general interference effects generating higher demands on attentional and cognitive control. In particular, prediction errors may render irrelevant features more salient and generate attentional capture that interferes with the ongoing task. For instance, interference could be based on a series of automatic and sequential attentional switches between salient feature dimensions before attention could then be deployed to the task-relevant dimension (spatial frequency). This interpretation corresponds to findings from visual search experiments, showing that search for a target with an unknown target-defining feature results in higher RT costs when the feature could change between different dimensions (e.g., color and orientation) compared to features within the same dimension (e.g., red and blue) (Müller et al. 1995; Treisman 1988). Alternatively, attentional capture by irrelevant but salient stimulus features may bind attentional resources on irrelevant feature dimensions and will, hence, decrease those available for the task performed.

In summary, the present results suggest that feature expectations for color and orientation are processed and resolved independently, and are unaltered by processes related to object binding. This finding is consistent with an early implementation of predictive coding within separate feature channels. Although the present findings cannot rule out that prediction errors for different object features might possibly be combined into an object-level expectancy, our results do not support the view that object feature binding leads to mutual influences of predictions errors of different object features.
